# Supplementary material for: Irradiation of UVC LED at 277 nm inactivates coronaviruses in association to photodegradation of spike protein
Source: Heliyon. 2022 Oct 17;8(10):e11132. doi: 10.1016/j.heliyon.2022.e11132 (PMC9575548; doi:10.1016/j.heliyon.2022.e11132)
Supplement: 20220926_SuppFig_277nm [file mmc1.docx]

**Supplementary Figures**

**Figure S1.** Wavelength spectrum of different UVC light sources


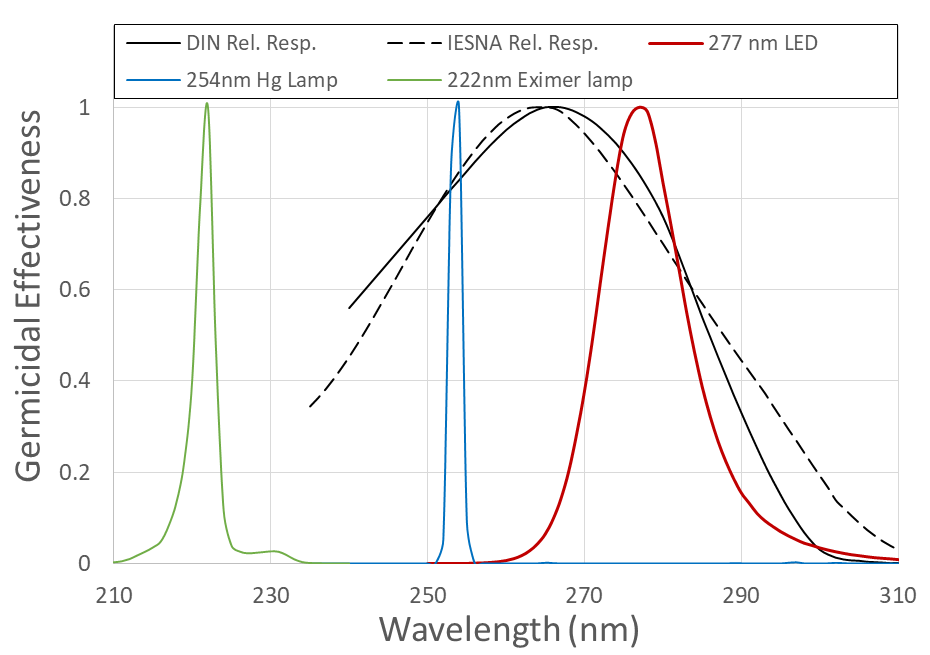


**Figure S2.** Schematic diagram of UVC enclosure

Light Source

Opening

UVC Light Source

Enclosure

Spectroradiometer

Opening

Petri dish

Door

Working

Distance

**Figure S3** Full non-adjusted images appeared in the paper

For Figure 3B bottom panel,


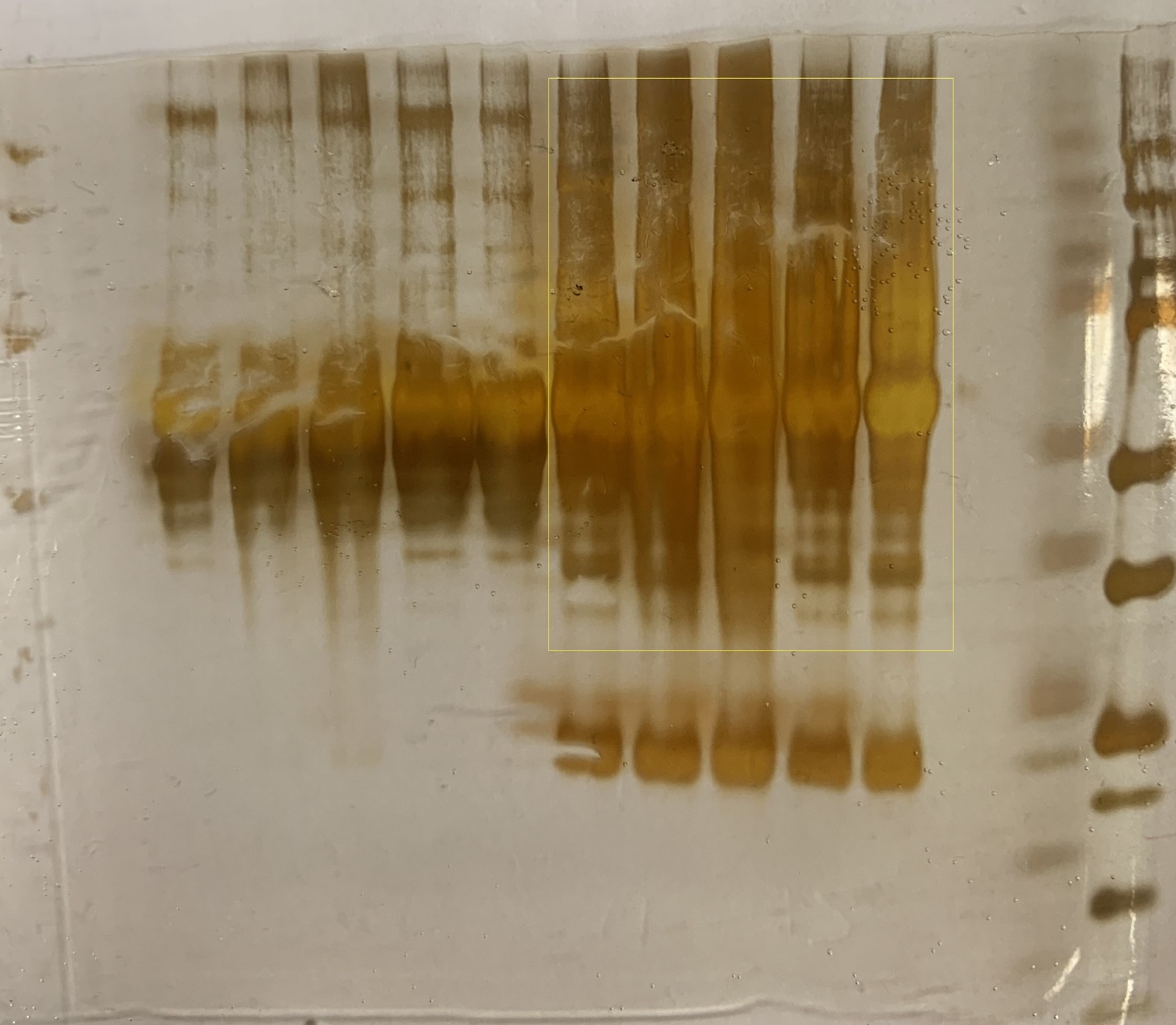


50

37

75

100

150

250

kDa

For Figure 3B top panel,


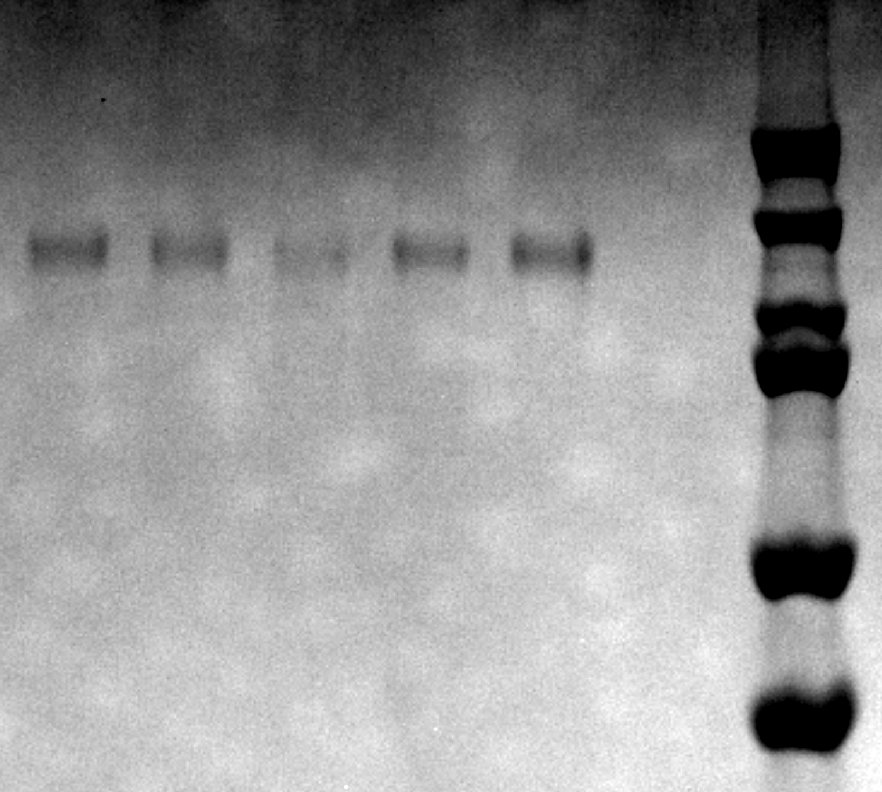


kDa

100

150

250

For Figure 3C,


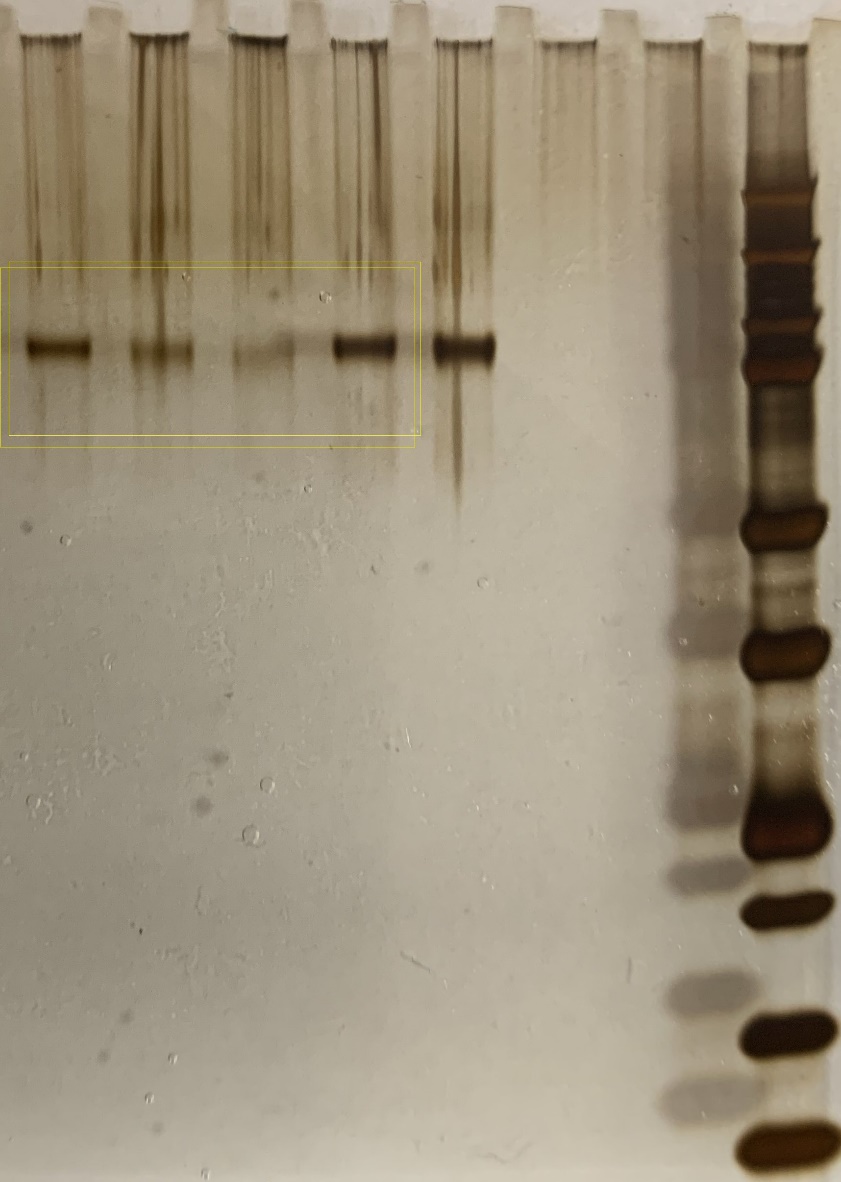


75

100

150

kDa

250

For Figure 4A,


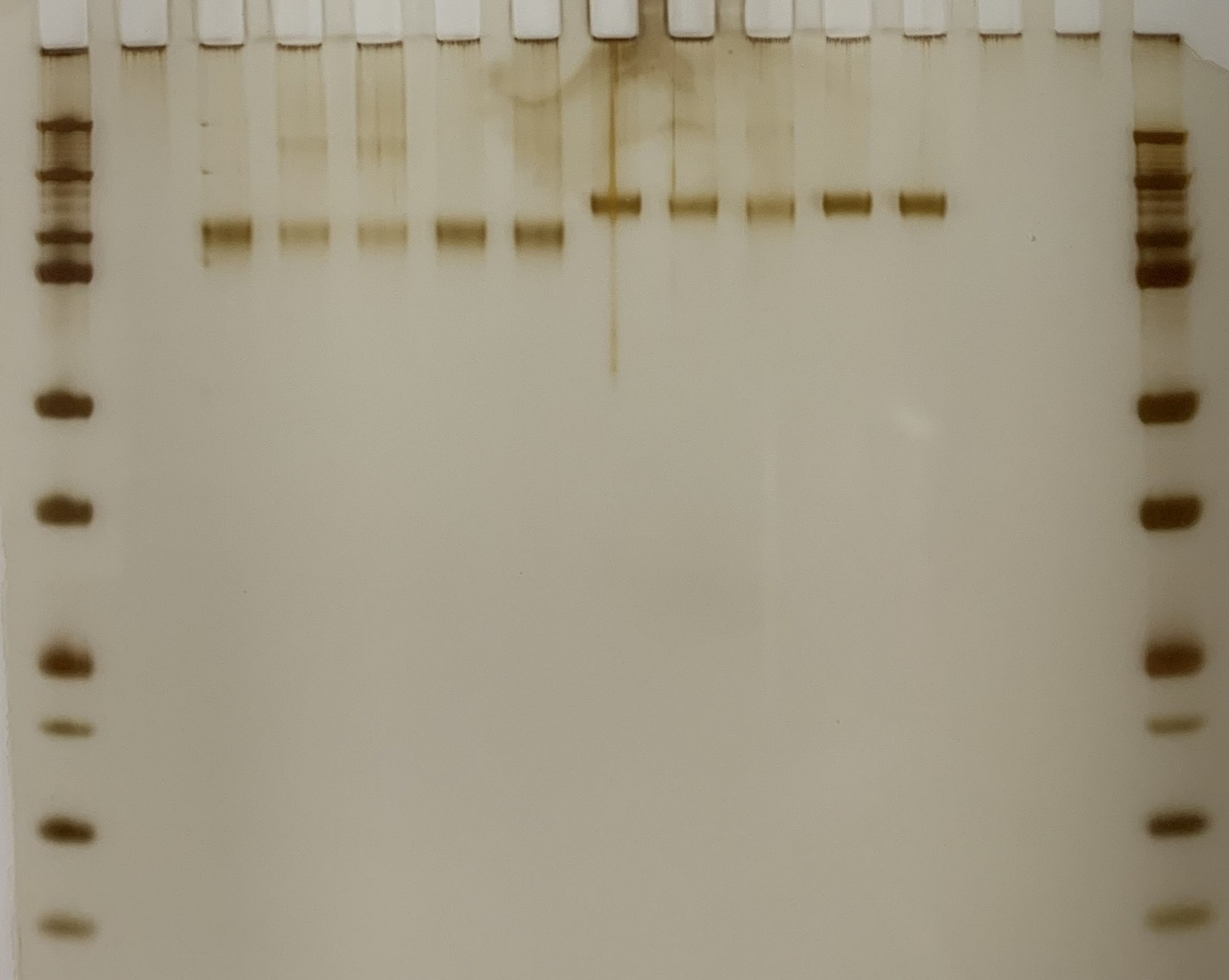


75

100

kDa

150

250

For Figure 4C, lower exposure


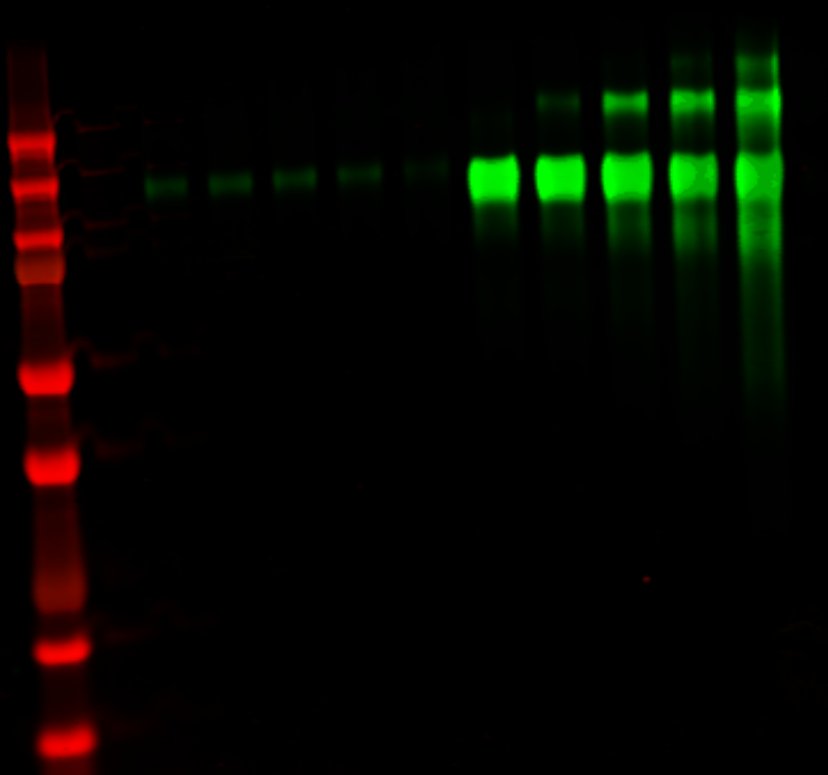


75

kDa

100

150

250

For Figure 4C, high exposure


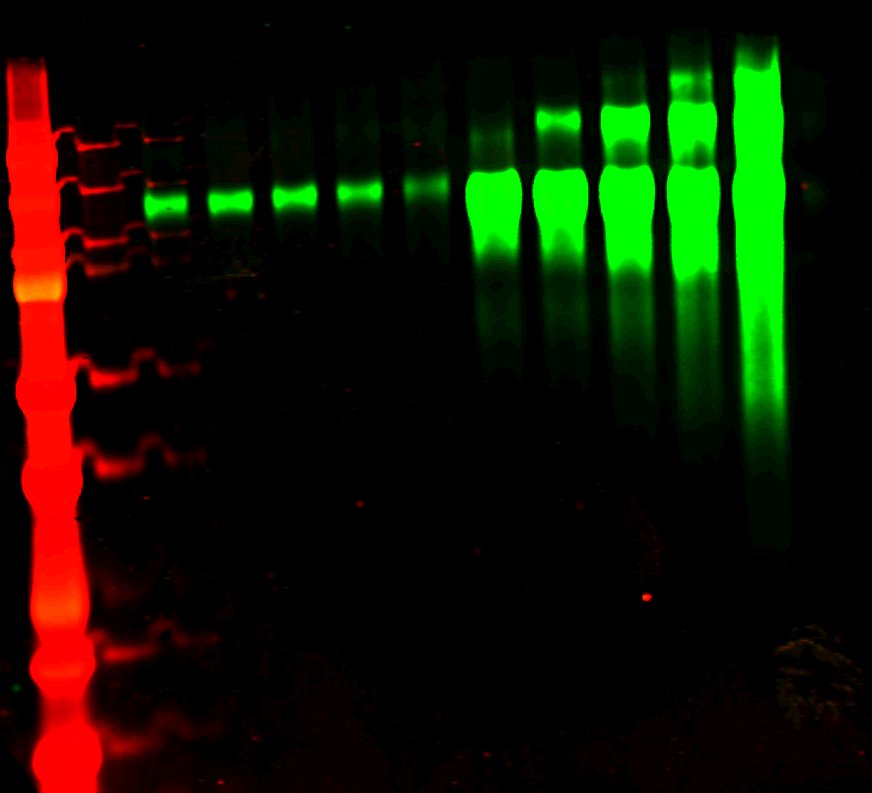


For Figure 4E,


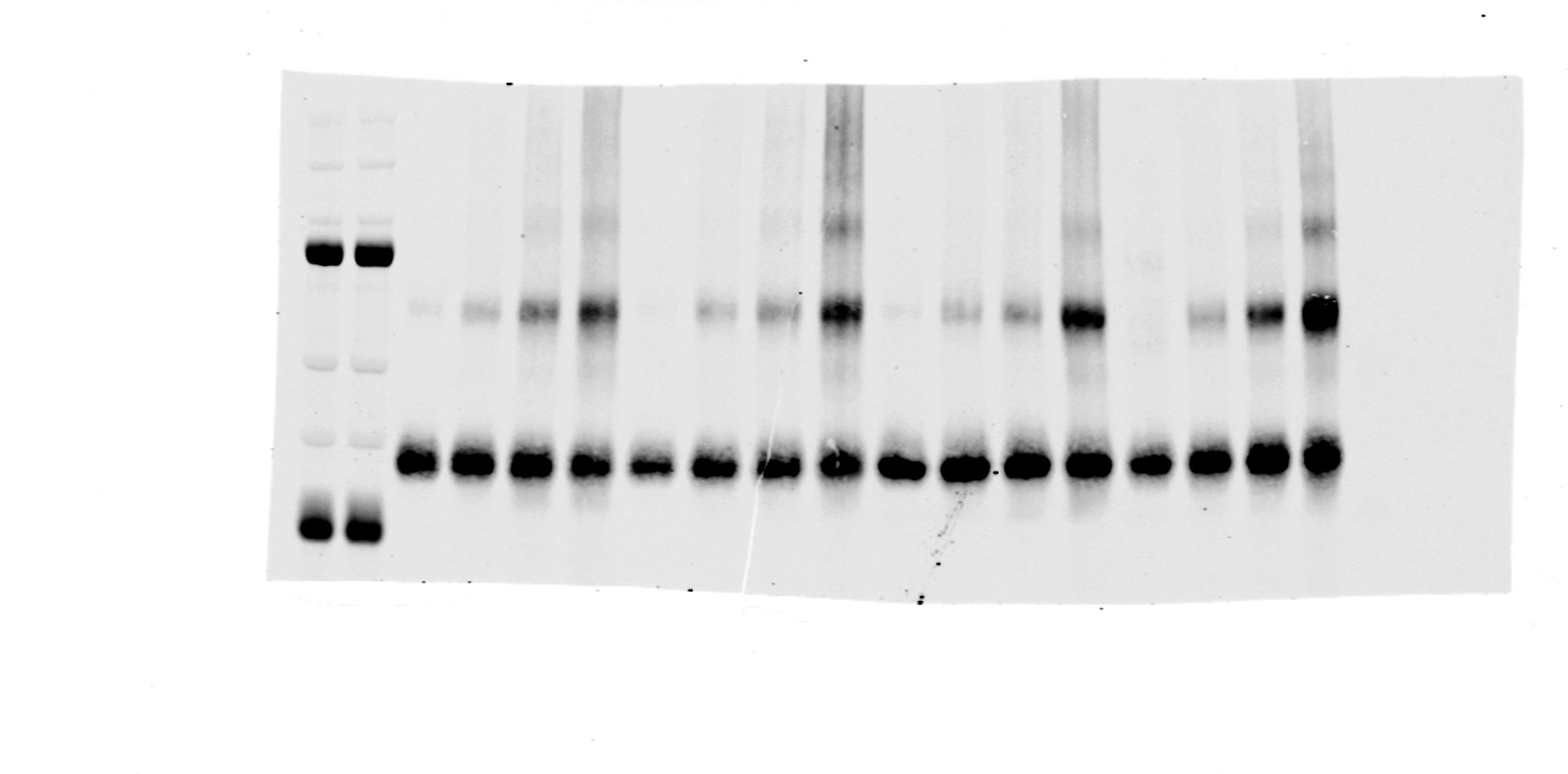


37

50

75

250

150

100

kDa

**Table S1.** Two-way ANOVA results for hCoV-OC43 infectivity curves.

|  |  |  |  |  |  |
| --- | --- | --- | --- | --- | --- |
| **Source of Variation** | **% of total variation** | **P value** | **P value summary** | **Significant?** | **Geisser-Greenhouse's epsilon** |
| Row Factor^a^ x Column Factor^b^ | 9.183 | <0.0001 | **** | Yes |  |
| Row Factor | 85.96 | <0.0001 | **** | Yes | 0.4027 |
| Column Factor | 4.361 | 0.0001 | *** | Yes |  |
| Subject | 0.2266 | 0.0628 | ns | No |  |
|  |  |  |  |  |  |
| **ANOVA table** | **SS** | **DF** | **MS** | **F (DFn, DFd)** | **P value** |
| Row Factor x Column Factor | 6035 | 6 | 1006 | F (6, 18) = 100.7 | P<0.0001 |
| Row Factor | 56488 | 3 | 18829 | F (1.208, 7.249) = 1884 | P<0.0001 |
| Column Factor | 2866 | 2 | 1433 | F (2, 6) = 57.75 | P=0.0001 |
| Subject | 148.9 | 6 | 24.81 | F (6, 18) = 2.483 | P=0.0628 |
| Residual | 179.9 | 18 | 9.992 |  |  |
|  |  |  |  |  |  |
| **Data summary** |  |  |  |  |  |
| Number of columns (Column Factor) | 3 |  |  |  |  |
| Number of rows (Row Factor) | 4 |  |  |  |  |
| Number of subjects (Subject) | 9 |  |  |  |  |

^a^ Row factor refers to different timings of UVC exposure.

^b^ Column factor refers to the different UVC wavelengths utilized.

**Table S2.** Two-way ANOVA results for hCoV-229e infectivity curves.

|  |  |  |  |  |  |
| --- | --- | --- | --- | --- | --- |
| \| **Source of Variation** \| **% of total variation** \| **P value** \| **P value summary** \| **Significant?** \| **Geisser-Greenhouse's epsilon** \| \| --- \| --- \| --- \| --- \| --- \| --- \| \| Row Factor x Column Factor \| 6.090 \| <0.0001 \| **** \| Yes \|  \| \| Row Factor \| 87.85 \| <0.0001 \| **** \| Yes \| 0.3988 \| \| Column Factor \| 5.446 \| <0.0001 \| **** \| Yes \|  \| \| Subject \| 0.07287 \| 0.8687 \| ns \| No \|  \| \|  \|  \|  \|  \|  \|  \| \| **ANOVA table** \| **SS** \| **DF** \| **MS** \| **F (DFn, DFd)** \| **P value** \| \| Row Factor x Column Factor \| 3633 \| 6 \| 605.5 \| F (6, 18) = 33.51 \| P<0.0001 \| \| Row Factor \| 52401 \| 3 \| 17467 \| F (1.196, 7.178) = 966.6 \| P<0.0001 \| \| Column Factor \| 3249 \| 2 \| 1624 \| F (2, 6) = 224.2 \| P<0.0001 \| \| Subject \| 43.47 \| 6 \| 7.245 \| F (6, 18) = 0.4009 \| P=0.8687 \| \| Residual \| 325.3 \| 18 \| 18.07 \|  \|  \| \|  \|  \|  \|  \|  \|  \| \| **Data summary** \|  \|  \|  \|  \|  \| \| Number of columns (Column Factor) \| 3 \|  \|  \|  \|  \| \| Number of rows (Row Factor) \| 4 \|  \|  \|  \|  \| \| Number of subjects (Subject) \| 9 \|  \|  \|  \|  \| \| Number of missing values \| 0 \|  \|  \|  \|  \| |  |  |  |  |  |

^a^ Row factor refers to different timings of UVC exposure.

^b^ Column factor refers to the different UVC wavelengths utilized.
